# Combination effect of lapatinib with foretinib in HER2 and MET co-activated experimental esophageal adenocarcinoma

**DOI:** 10.1038/s41598-019-54129-7

**Published:** 2019-11-26

**Authors:** Md. Sazzad Hassan, Fiona Williams, Niranjan Awasthi, Margaret A. Schwarz, Roderich E. Schwarz, Jun Li, Urs von Holzen

**Affiliations:** 1 Department of Surgery, Indiana University School of Medicine, South Bend, IN 46617 USA; 2 Department of Biological Sciences, University of Notre Dame, Notre Dame, IN 46556 USA; 3 Goshen Center for Cancer Care, Goshen, Goshen, IN 46526 USA; 4 Harper Cancer Research Institute, South Bend, IN 46617 USA; 5 Department of Pediatrics, Indiana University School of Medicine, South Bend, IN 46617 USA; 6 University of Basel, Basel, Switzerland; 7 Department of Applied and Computational Mathematics and Statistics, University of Notre Dame, Notre Dame, IN 46556 USA

**Keywords:** Cancer therapeutic resistance, Oesophageal cancer

## Abstract

Recent studies have demonstrated that HER2 and MET receptor tyrosine kinases are co-overexpressed in a subset esophageal adenocarcinoma (EAC). We therefore studied the usefulness of combining HER2 and MET targeting by small-molecule inhibitors lapatinib and foretinib, respectively, both in *in-vitro* and *in-vivo* models of experimental EAC. We characterized MET and HER2 activation in a panel of human EAC cell lines, and the differential susceptibility of these EAC cell lines to single agent or combination of foretinib and lapatinib. We then explored the antitumor efficacy with survival advantage following foretinib and lapatinib monotherapy and in combination in murine subcutaneous xenograft and peritoneal metastatic survival models of human EAC. The OE33 EAC cell line with strong expression of phosphorylated both MET and HER2, demonstrated reduced sensitivity to foretinib and lapatinib when used as a single agent. The co-administration of foretinib and lapatinib effectively inhibited both MET and HER2 phosphorylation, enhanced inhibition of cell proliferation and xenograft tumor growth by inducing apoptosis, and significantly enhanced mouse overall survival, overcoming single agent resistance. In the OE19 EAC cell line with mainly HER2 phosphorylation, and the ESO51 EAC cell line with mainly MET phosphorylation, profound cell growth inhibition with induction of apoptosis was observed in response to single agent with lack of enhanced growth inhibition when the two agents were combined. These data suggest that combination therapy with foretinib and lapatinib should be tested as a treatment option for HER2 positive patients with MET-overexpressing EAC, and could be a novel treatment strategy for specific EAC patients.

## Introduction

The two main subtypes of esophageal cancer are esophageal squamous cell-carcinoma and esophageal adenocarcinoma^1^. While esophageal squamous cell-carcinoma still accounts for 90 percent of the cases of esophageal cancer worldwide, esophageal adenocarcinoma has become the dominant type in the United States and the Western world, and the number of cases is ever increasing^2–5^. The overall 5 year survival rate of EAC is below 20 percent, and the prognosis for EAC remains poor even with modern combination therapies due to development of high resistance to chemotherapy^6,7^, and despite recent advances in surgical and radiation techniques as well as in systemic treatment options^6,8^. Moreover, 50–60 percent of EAC are unresectable at the time of diagnosis^9^. Although EAC seems to respond well initially to conventional chemotherapy, clinical benefit is limited and most patients eventually die from distant metastatic disease^10^. Therefore, new therapeutic approaches are urgently needed.

A major target for esophageal cancer therapies is the human epidermal growth factor receptor 2 (HER2)^11^. A subset of EAC has been shown to overexpress HER2. Trastuzumab (Herceptin), a monoclonal antibody to HER2 is currently the only FDA approved targeted therapy used for HER2 positive metastatic EAC^12^. In EAC models, there has been limited examination of HER-2 targeted agents. Unfortunately, HER2 positive advanced EAC patients frequently develop resistance to Herceptin through mechanisms still poorly understood^13^. An alternative anti-HER2 strategy has been the use of small-molecule tyrosine kinase inhibitors that target not only HER2 but other HER family proteins. Lapatinib is a potent ATP-competitive inhibitor that simultaneously inhibits both EGFR and HER2. However, lapatinib, a dual EGFR and HER2 inhibitor, has shown disappointing results in clinical trials of metastatic EAC^14^, and the mechanisms that contribute to lapatinib resistance are unknown. The resistance to lapatinib in EAC may be related to MET-EGFR crosstalk but lacks supportive *in-vivo* data. Foretinib is a small-molecule kinase inhibitor that inhibits cellular hepatocyte growth factor (HGF)-induced c-MET phosphorylation and prevents HGF-induced response to tumor cells^15,16^. Recent studies indicated that EAC is driven by amplification of c-MET and HER2 in a subset of patients who may be resistant to lapatinib therapy^17,18^. HER2 and MET overexpression is highly prevalent (20 to 30%) in EAC^18,19^ and HER2-MET co-overexpression is also frequent in EAC^20^. Thus lapatinib and foretinib combination therapy could be a novel strategy for treating EAC with overexpression/activation of MET and HER2.

In this study, we therefore hypothesized that MET activation may lead to lapatinib resistance in HER2-driven esophageal adenocarcinoma, and tested the feasibility of MET targeting by small-molecule inhibitor Foretinib in EAC cells. We present for the first time the *in-vivo* administration of lapatinib with foretinib for treating experimental EAC.

## Materials and Methods

### Cell lines, cell culture and reagents

Human esophageal adenocarcinoma cell lines (ESO26, OE33, ESO51, SK-GT-2, OE19, OACM5.1 C and Flo-1) were obtained from Sigma Aldrich (St. Lois, MO). All cell lines except Flo-1 were cultured in RPMI-1640 medium (Gibco, Grand Island, New York, USA) whereas Flo-1 was cultured in DMEM medium (Gibco) supplemented with 10% fetal bovine serum (Hyclone), 2 mM GlutaMax (Gibco), 100 U/ml penicillin, 100 mg/ml streptomycin at 37 °C in a humidified atmosphere of 95% air – 5% CO_2_. Lapatinib and Foretinib were purchased from LC labs (Woburn, MA). Paclitaxel and Carboplatin were obtained from local pharmacy. The cell proliferation reagent WST-1 was purchased from Roche Diagnostic Corporation (Indianapolis, IN).

Lapatinib-resistant OE19 (LPR-OE19) cells were established from OE19 cells by intermittent exposure to increasing concentrations of lapatinib for a period of five months. Briefly, aliquots of OE19 cells in the exponential growth phase were seeded into 25 cm^2^ culture flasks. Lapatinib (10 μM) was added for 48 hours during the mitotic phase, and then the cells were transferred into drug-free culture medium for around 15 days until the cells reached 80% confluency, after which lapatinib was added for the next 48 hours at twice the previous concentration. We continued this process while observing cell death every day, changing to fresh complete culture medium, and performing drug sensitivity to lapatinib by WST-1 assay every month. This process was continued until the concentration of lapatinib in the medium reached 80 μM after around 150 days. Thus, lapatinib-resistant OE19 (LPR-OE19) cells were obtained.

### Cell viability assay

Cell viability was evaluated by the colorimetric WST-1 assay as previously described^21,22^. The measurement is based on the ability of viable cells to cleave the sulfonated tetrazolium salt WST-1 (4-[3-(4-iodophenyl)-2-(4-nitrophenyl)-2H-5-tetrazolio]-1,3-benzene disulfonate) by mitochondrial dehydrogenases. EAC cells (4,000 to 5,000 cells per well) were plated in a 96-well plate in regular growth medium containing 10% FBS. After 16 hours the medium was replaced with 2% FBS containing medium and the cells were treated with lapatinib, foretinib, paclitaxel or carboplatin alone or in combinations. After 72 hours, 10 μL WST-1 reagent was added in each well followed by additional incubation for 2 hours. The absorbance at 450 nm was measured using a microplate reader.

### Western blot analysis

Western blot analyses were determined as described by us previously^21,23,24^. Protein lysates were prepared by treating sub-confluent cells with lapatinib, foretinib alone or in combination (all 5 µM), and lysed after 16 hours for Western blotting. Cell lysates were prepared by scraping cells from culture plates in cold lysis buffer (20 mM HEPES, 150 mM NaCl, 1 mM EDTA, 0.5% Na^ +^ deoxycholate, 1% Nonidet P-40, and 1 mM DTT, pH 7.4) containing protease and phosphatase inhibitor cocktails (both from Sigma-Aldrich, St. Louis, MO). Protein lysates of subcutaneous tumors were prepared by snap freezing tumor tissues in liquid nitrogen and stored at –80 °C. These xenograft tissue samples were homogenized in a cold lysis buffer containing protease and phosphatase inhibitor cocktails using a glass dounce tissue homogenizer. Polyacrylamide gel electrophoresis was used to separate equal amounts of protein samples, which were then transferred to nitrocellulose membranes for analysis. The nitrocellulose membranes were blocked for 1 hour in PBS-T at room temperature and then incubated overnight at 4 °C with the following primary antibodies: cleaved poly (ADP-ribose) polymerase-1 (c-PARP) (Catalog #5625), cleaved caspase-3 (Catalog #5664), total MET (Catalog #8198) and phospho-MET(Catalog #3077), total HER2 (Catalog #2165) and phospho-HER2 (Catalog #2243) (all from Cell Signaling Technology, Beverly, MA) and β-actin (Sigma-Aldrich, St. Louis, MO) (Catalog #A1978). Blots were incubated with the corresponding HRP-conjugated secondary antibodies (Pierce Biotechnologies, Santa Cruz, CA) for 1 hour at room temperature. Specific bands were detected using the enhanced chemiluminescence reagent (ECL, Perkin Elmer Life Sciences, Boston, MA.) Protein bands were quantified using ImageJ software (National Institutes of Health).

### Subcutaneous tumor xenografts

All mouse experiments used in this study were carried out in accordance with the standards and guidelines of the Institutional Animal Care and Use Committee (IACUC) at the University of Notre Dame and confirmed to NIH guidelines. All animal research used in this study was approved by the University of Notre Dame IACUC under protocol 15-08-2631. Female athymic nude mice (4 to 6 weeks old) were subcutaneously injected with the OE33 EAC cell line (5 × 10^6^). Measurements of subcutaneous tumor size were started when the tumors reached an average volume of 50–60 mm^3^. All mice had measurable tumor two weeks after OE33 cell injection. The mice were then randomly grouped (n = 5 per group) and treated intraperitoneally as described earlier^21,22^ with vehicle, lapatinib (60 mg/kg, 5 times a week for 2 weeks), foretinib (30 mg/kg, 5 times a week for 2 weeks), paclitaxel (20 mg/kg, 2 times a week for 2 weeks) or carboplatin (50 mg/kg, 2 times a week for 2 weeks) alone or in combinations. The tumor size was measured twice a week for four weeks with slide calipers and tumor volume (TV) was calculated as (W^2^XL)/2, where W is width and L is length of the tumor^25^. Relative tumor volume (RTV) was calculated according to the following formula; RTV = TV_n_/TV_0_ where TV_n_ is the tumor volume at the day of measurement and TV_0_ is the tumor volume on the first day of measurement^26^. Mice weight was measured twice a week during the period of the study. At the end of experiments all mice were euthanized by CO2 using the Euthanex Euthanasia chamber according to University of Notre Dame IACUC-approved procedure. Mice were euthanized when turning moribund according to predefined criteria^27,28^. In all studies, mice were euthanized if they had rapid weight loss (>20%) or weight gain (>20% due to ascites), loss of ability to ambulate, presence of labored respiration, inability to drink or feed, lack of response to external stimuli, muscle atrophy or more than 2 cm tumor size in any direction and the actual death of the animal was not an anticipated endpoint in these experiments. At the end of the study tumors were removed, weighted, dissected and processed for histological, immunohistochemical and western blot analysis.

### Peritoneal-disseminated animal survival model

Animal survival studies were performed using female non-obese diabetic/severe combined immunodeficient (NOD/SCID) mice (4–6 weeks of age) as previously described^21,22^. Briefly, the mice were injected intraperitoneally with OE33 (10 × 10^6^) cells and two weeks after tumor cell injection, mice were randomized (n = 5 per group) to receive vehicle, lapatinib (60 mg/kg, 5 times a week for 2 weeks), foretinib (30 mg/kg, 5 times a week for 2 weeks), paclitaxel (20 mg/kg, 2 times a week for 2 weeks) or carboplatin (50 mg/kg, 2 times a week for 2 weeks) alone or in combinations. Animal survival was evaluated from the first day of treatment until death.

### Immunofluorescence analysis

Immunofluorescence was performed on histological sections of 4% paraformaldehyde-fixed OE33 tumor xenografts. Paraffin embedded tissue blocks were cut into 5 µM tissue sections, deparaffinized and rehydrated. The tissue sections were incubated with a 1:200 dilution of the Ki67 antibody (ab15580, Abcam, Cambridge, MA) and the cleaved caspase 3 antibody (#9661, Cell Signaling Technology, Beverly, MA), followed by incubation with a 1:200 dilution of an anti-rabbit-Cy3 secondary antibody (Jackson ImmunoResearch Laboratories, West Grove, PA). Slides were mounted using a mounting solution containing 4′,6-diamidino-2-phenylindole (DAPI) (Invitrogen, Carlsbad, CA). Fluorescence microscopy was used to detect fluorescent signals. The intratumoral proliferative and apoptotic index were determined by calculating the Ki67 and cleaved caspase 3 positive cells from five different high-power fields (HPF) in a blinded manner in each group.

### Immuno-paired-antibody detection or ActivSignal assay

OE19 and LPR-OE19 cells were then subjected to immune-paired antibody detection (IPAD) as described on https://www.activsignal.com/service/. The IPAD or ActivSignal assay (ActiveSignal, LLC, Natick, MA) examines phosphorylation or expression of 70 different human protein targets, which cover 20 major signaling pathways^29^. The ActiveSignal assay uses paired antibodies for each target protein and detection occurs only if both antibodies in a pair bind to a specific target protein. The detection of the paired antibodies is facilitated via a special DNA barcode conjugated to antibodies, which were quantified using Next Generation Sequencing or the Fluidigm digital PCR platform.

### Statistical analysis

*In vitro* cell proliferation, proliferative and apoptotic index data were expressed as mean ± standard deviation. Statistical analysis was performed by ANOVA for multiple group comparison and Student’s t-test for the individual group comparison. The comparison of survival time between different groups was done by using the log-rank test^21^ using GraphPad Prism 7.0 Software (GraphPad Software, San Diego, CA). The comparison of the relative tumor volume (RTV) between treatment groups was done by first normalizing the RTV values at day 14 by the mean TRV value of the corresponding group at day 0, and then applying the two-sample t test, implemented in the “t.test” R function. p < 0.05 was considered statistically significant.

## Results

### Effect of lapatinib and foretinib on human EAC cell growth inhibition

**Figure 1:**
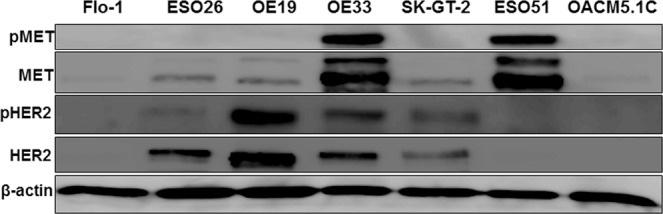
Dual phosphorylation of MET and HER2 in OE33 esophageal adenocarcinoma cell line. Protein lysates were collected from seven human esophageal adenocarcinoma cell lines and analyzed for phospho MET (pMET), MET, phospho HER2 (pHER2) and HER2 expression. β-actin serves as loading control. OE33 human esophageal adenocarcinoma cell line showed strong phosphorylation of both MET and HER2.

**Figure 2:**
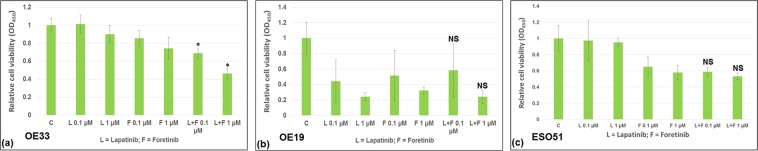
OE33 cells showed enhanced inhibition of cell proliferation when lapatinib and foretinib were co-administered. Human esophageal adenocarcinoma (**a**) OE33 (**b**) OE19 and (**c**) ESO51 cells were plated on 96-well plates and treated with 0.1 and 1 µM of lapatinib and foretinib alone or in combinations. After 72 hours, 10 µl WST-1 reagent was added in each well and incubated for 2 additional hours. The absorbance at 450 nm was measured using a microplate reader. The resulting number of viable cells was calculated by measuring absorbance of color produced in each well. Results shown were representative of three independent experiments with 6 parallel wells (mean ± SDE). * (indicates p < 0.05) represents combination treatment is significantly different from the same dose of lapatinib (**L**) or foretinib (**F**) treatment alone (**a**). NS represents that the differences are non-significant from the same dose of lapatinib (**b**) or foretinib (**c**).

To find the MET and HER2 activation status, we evaluated levels of their phosphorylation in a panel of seven EAC cell lines (Fig. 1). We found that the OE33 EAC cell line showed very strong phosphorylation of both MET and HER2, whereas OE19 cells showed phosphorylation of mainly HER2, and ESO51 showed phosphorylation of largely MET. Interestingly, OE33 EAC cells with MET and HER2 co-activation were resistant to single-agent MET-targeting foretinib (0.1 μM), and HER2-targeting lapatinib (1 μM) induced inhibition of cell growth, but showed significantly enhanced cell growth inhibition when foretinib and lapatinib were co-administered (Fig. 2a). Contrary to that, OE19 EAC cells with mainly HER2 phosphorylation showed significant cell growth inhibition with single-agent HER2-targeting lapatinib at 0.1 μM, and co-administration of lapatinib with foretinib didn’t further enhance OE19 cell growth inhibition (Fig. 2b). On the other hand, ESO51 cells with predominately MET phosphorylation showed significant cell growth inhibition with single-agent MET-targeting foretinib at 0.1 μM, and co-administration of lapatinib with foretinib didn’t further enhance ESO 51 cell growth inhibition (Fig. 2c).

**Figure 3:**
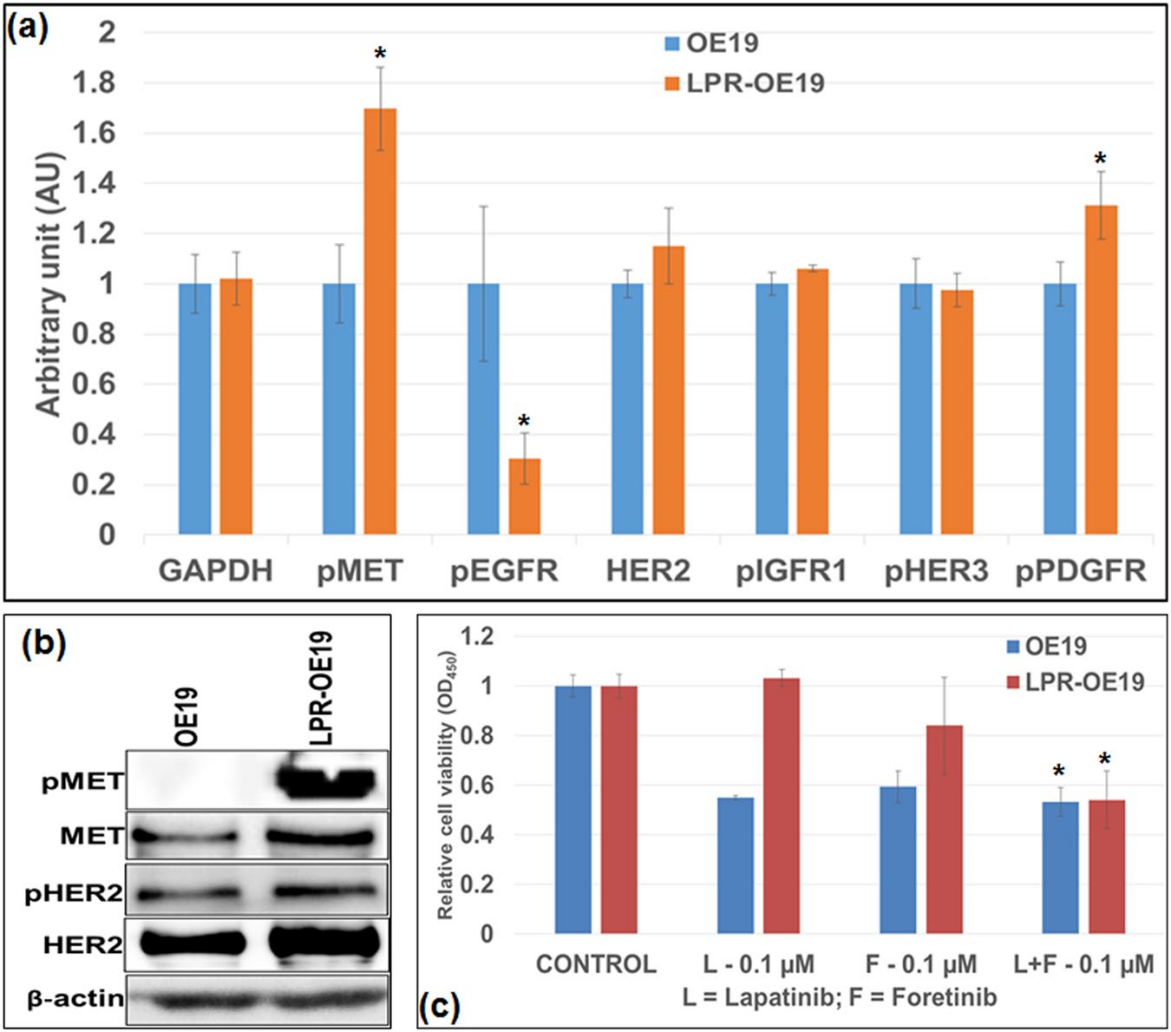
Lack of MET activation rendered OE19 cells sensitive to single-agent lapatinib induced inhibition of cell proliferation whereas MET activation rendered LPR-OE19 cells resistant to lapatinib induced inhibition of cell proliferation. (**a**) OE19 and LPR-OE19 cells were plated in 96-well plates in triplicates and subjected to ActiveSignal Assay analysis, which measures expression or activation of 70 proteins. The graph shows the major tyrosine kinase proteins involved in lapatinib resistance mechanism. (**b**) Whole cell lysates of OE19 and LPR-OE19 were subjected to western blot analysis using pMET, MET, pHER2, HER2 and β-actin antibodies. (**c**) OE19 and LPR-OE19 cells were plated on 96-well plates and treated with 0.1 µM of lapatinib and foretinib alone or in combinations. After 72 hours, 10 µl WST-1 reagent was added in each well and incubated for 2 additional hours. The absorbance at 450 nm was measured using a microplate reader. Results shown were representative of three independent experiments with 6 parallel wells (mean ± SDE). * (p > 0.05) indicates L + F 0.1 µM versus L 0.1 µM or F 0.1 µM or control in OE19 and L + F 0.1 µM versus control in LPR-OE19.

We also tested MET and HER2 activation status and sensitivity to their inhibitors in newly generated lapatinib resistant OE19 (LPR-OE19) EAC cells. Interestingly, LPR-OE19 cells showed significant upregulated expression of phosphorylated MET compared to parent OE19 cells, detected by both Activesignal assay (Supplementary Fig. S1 and Fig. 3a) and western blot analysis (Fig. 3b). In addition, LPR-OE19 cells showed significantly reduced sensitivity to lapatinib compared to parent OE19 cells, and the co-administration of lapatinib and foretinib significantly enhanced inhibition of cell proliferation in LPR-OE19 cells (Fig. 3c).

### *In-vitro* effect of lapatinib and foretinib on HER2 and MET signaling and the expression of apoptosis markers cleaved PARP and caspase 3

**Figure 4:**
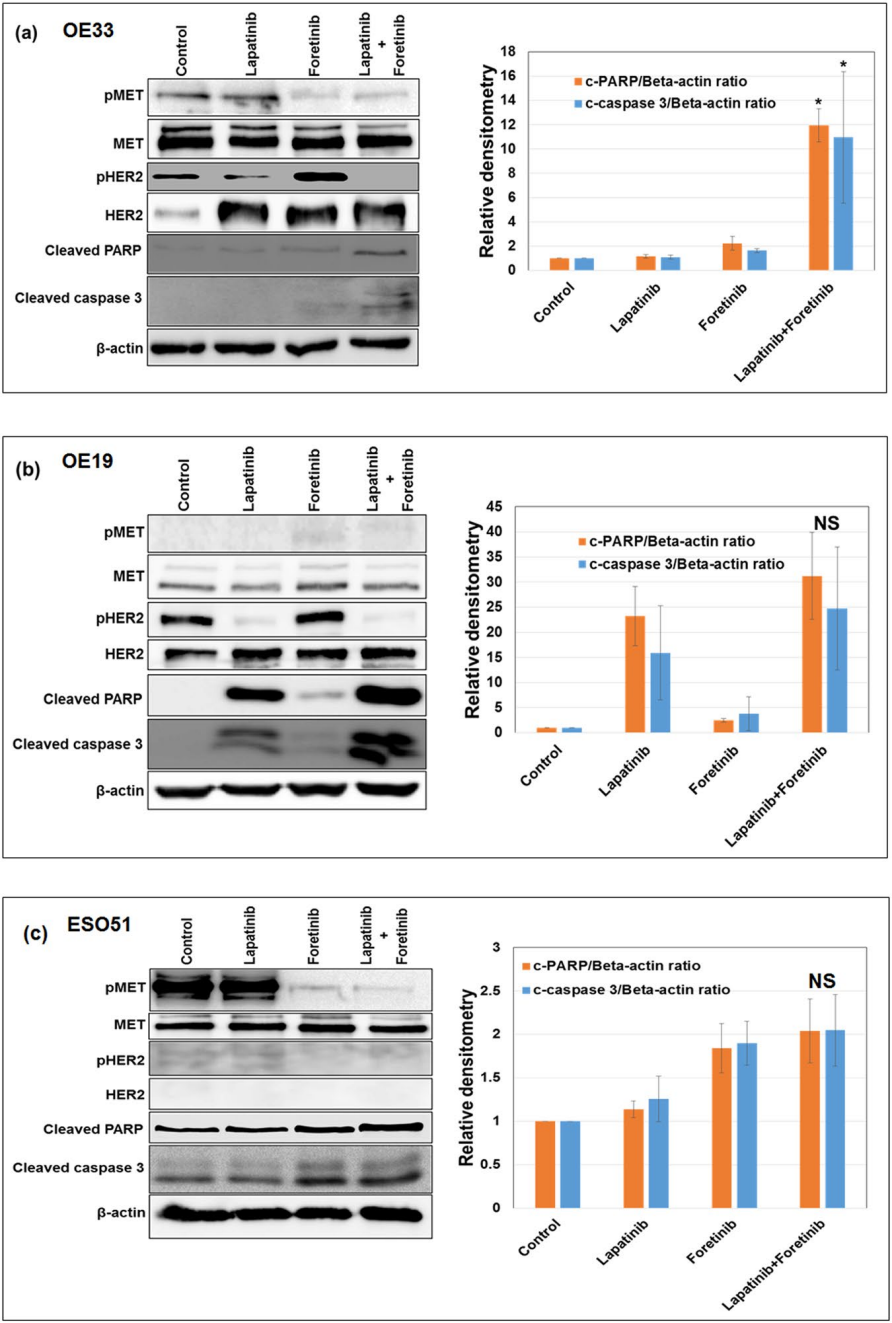
OE33 cells showed enhanced apoptosis when lapatinib and foretinib were co-administered. Sub-confluent monolayer of human esophageal adenocarcinoma (**a**) OE33 (**b**) OE19 and (**c**) ESO51 cells were treated with 1 μM of lapatinib and foretinib alone or in combination for 16 hours. Total cell extracts were analyzed by western blots with antibodies to phospho MET, MET phospho HER2, HER2, cleaved poly (ADP-ribose) polymerase-1 (cleaved PARP), cleaved caspase 3, and β-actin. The intensity of bands of cleaved PARP and cleaved caspase 3 was quantitated by densitometry using imageJ software and is represented in the bar graph after normalizing values with β-actin. Results shown were representative of three independent experiments. * (p < 0.05) represents combination treatment is significantly different (**a**) from control and the same dose of lapatinib or foretinib treatment alone (n = 3). NS represents that the differences are non-significant from lapatinib (**b**) and from foretinib (**c**) (n = 3).

Immunoblot analysis to determine the effect of lapatinib on HER signaling and foretinib on MET signaling revealed that lapatinib blocked the expression of phospho-HER2 (pHER2), and foretinib blocked the expression of phospho-MET (pMET) (Fig. 4). Expression of apoptosis related proteins cleaved PARP-1 and cleaved caspase-3 showed significantly higher levels after combined treatment of 1 μM of lapatinib and foretinib, as compared to that of single agent treatments in OE33 cells (Fig. 4a). In OE19 cells, treatment of 1 μM of lapatinib alone produced strong expression of cleaved PARP and caspase 3 with less enhancement when lapatinib was combined with 1 μM of foretinib, compared to that effect observed in OE33 cells (Fig. 4b). Similarly, in ESO51 cells, 1 μM of foretinib treatment alone produced cleavage of PARP and caspase 3 with less enhancement when foretinib was combined with 1 μM of lapatinib (Fig. 4c), compared to that effect observed in OE33 cells.

### Effect of lapatinib and foretinib treatments on human EAC xenograft growth

**Figure 5:**
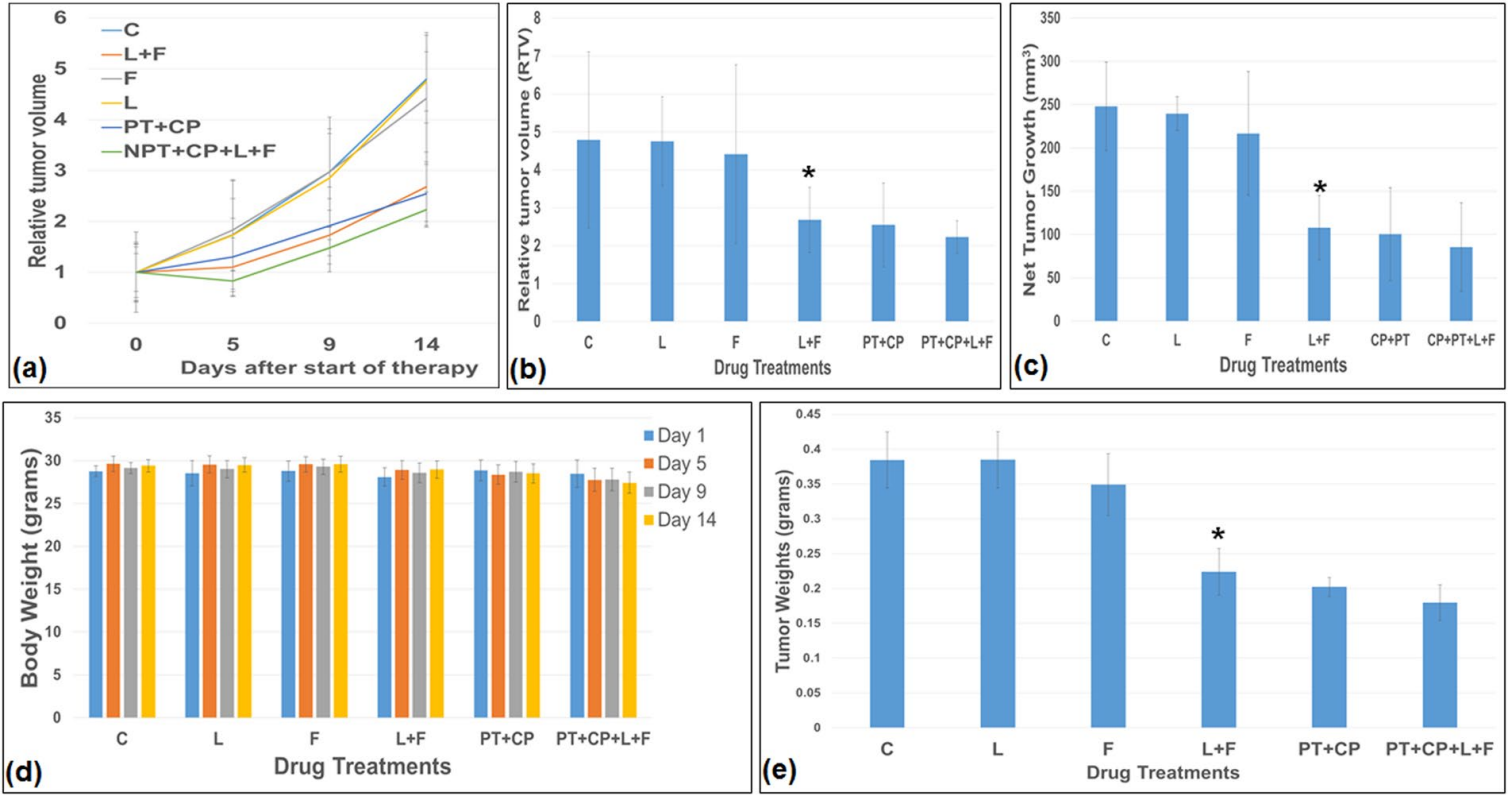
Antitumor activity of lapatinib (L) and foretinib (F) in OE33 tumor xenografts. OE33 cells were subcutaneously injected in nude mice and treated with lapatinib (L), foretinib (F) either alone or in combinations with carboplatin (CP) and paclitaxel (PT). (**a,b**) Relative tumor volume (RTV) was calculated by dividing the tumor volume at any time by the tumor volume at the start of treatment. (**a**) RTV changes over a period of 4 weeks after subcutaneous injection of 5 × 10^6^ OE33 cells. (**b**) RTV changes after drug treatments were compared. (**c**) Net tumor growth was calculated by subtracting tumor volume on the first treatment day from that on the final day. Data are representative of mean values ± standard deviation from 5 mice per group. (**d**) No significant body weight change was observed after drug treatments compared to control in the OE33 subcutaneous mouse model. (**e**) Tumor weight changes after drug treatments were compared. * indicates p < 0.05 versus control C or lapatinib L or foretinib F.

We then determined the *in-vivo* antitumor efficacy of lapatinib and foretinib alone and in combinations in a murine xenograft model using OE33 cells. We also determined the antitumor effect of lapatinib and foretinib with standard paclitaxel/carboplatin chemotherapeutic combination. Relative tumor volumes (RTV), net tumor growth, tumor weight and response to treatment groups are shown in Fig. 5. Lapatinib (60 mg/kg, 5 times a week for 2 weeks) and foretinib (30 mg/kg, 5 times a week for 2 weeks) were well tolerated without obvious signs of toxicity as judged by mouse weight (Fig. 5d) and daily assessment. Lapatinib in combination with foretinib treatment resulted in significantly reduced RTV, net tumor growth and tumor weight compared to those with foretinib or lapatinib treatment alone (Fig. 5b,c, and e). In subcutaneous xenografts using OE33 cells, average net tumor growth after two weeks in the different therapy groups was 247.83 mm^3^ in control, 216.71 mm^3^ after foretinib (p = 0.49), 239.68 mm^3^ after lapatinib (p = 0.74), and 108.06 mm^3^ after foretinib plus lapatinib (p = 0.0011). Net tumor growth inhibition in lapatinib (L), foretinib (F), L + F, CP (carboplatin) + PT (paclitaxel), CP + PT + L + F groups was 3%, 12.5%, 56.3%, 59.4%, and 65.5% as compared with the control. Although both lapatinib (3%, p = 0.74) and foretinib (12.5%, p = 0.45) alone were ineffective in reducing primary tumor growth, the growth inhibition rate was significantly higher when lapatinib was combined with foretinib (56.3%, p = 0.001), supporting the higher efficacy of the lapatinib plus foretinib regimen. Further enhancement in reducing primary tumor growth was observed (65.5%, p = 0.001) when lapatinib plus foretinib was combined with the standard chemotherapeutic regimen carboplatin (CP) plus paclitaxel (PT). In addition, compared to control, lapatinib (0.38426 g vs. 0.3847 g, p = 0.9867) and foretinib (0.38426 g vs. 0.3891 g, p = 0.22684) didn’t significantly decrease mean tumor weight as monotherapy but significantly decreased mean tumor weight as combination therapy (0.38426 g vs. 0.22347 g, p = 0.00013) with further enhancement in reducing mean tumor weight when lapatinib plus foretinib was combined with CP plus PT (0.22347 g vs. 0.1795 g, p = 0.0466) (Fig. 5e).

### *In-vivo* effect of lapatinib and foretinib treatment on HER2/MET phosphorylation and the expression of apoptosis markers cleaved PARP and caspase 3

**Figure 6:**
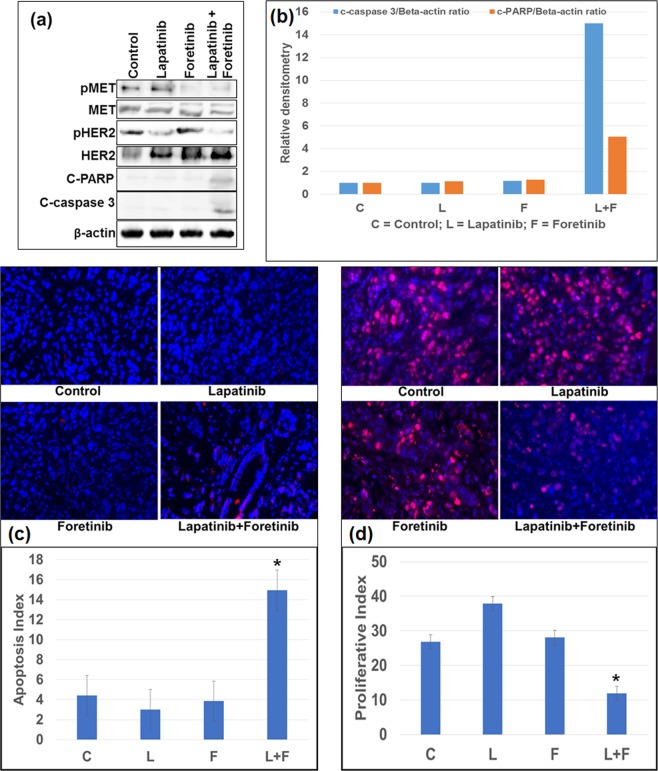
Higher pro-apoptotic and anti-proliferative *in-vivo* potency of lapatinib plus foretinib over lapatinib or foretinib alone. (**a**) *in-vivo* comparative effects of Lapatinib and Foretinib on the expression of apoptosis related proteins in OE33 esophageal adenocarcinoma xenografts. Tumor lysates were prepared from OE33 xenograft tumor tissue samples obtained from tumor bearing mice after Lapatinib or Foretinib monotherapy or combination therapy. Tumor lysates were then analyzed by immunoblotting with antibodies to phospho MET (pMET), MET, phospho HER2 (pHER2), HER2 cleaved poly (ADP-ribose) polymerase 1 (cleaved PARP), cleaved caspase 3 (C-caspase 3) and β-actin. (**b**) The intensity of C-caspase 3 and C-PARP was quantified by densitometry and represented in the bar graph after normalizing values with β-actin expression. Data are representative of pooled lysates obtained from tumors of 5 mice in each therapy group. (**c**) Intratumoral apoptosis was measured by staining tumor tissue sections with cleaved caspase 3. Cleaved caspase 3 positive apoptotic cells were counted in five different high power fields. (**d**) Intratumoral proliferation was measured by immunostaining tissue sections for Ki67 nuclear antigen. Ki67-positive cells were counted in five different high power fields. For both immunostaining experiments, slides were photographed under a fluorescence microscope and the data are expressed as the mean ± standard deviation. * Indicates p < 0.05 in L + F versus control, L or F.

The *in-vivo* effect of lapatinib and foretinib on HER2 and MET phosphorylation was investigated using the OE33 xenograft mouse model. A significant decrease in the expression of phospho HER2 and phospho MET was observed in the lapatinib and foretinib treated groups respectively (Fig. 6a). Evaluation of intratumoral apoptosis by analyzing the expression of cleaved caspase 3 (C-caspase 3) and PARP (C-PARP) in OE33 xenograft tumor pooled lysates revealed significant increase after *in-vivo* treatment with lapatinib plus foretinib compared to control, lapatinib or foretinib treatment alone (Fig. 6a,b). The apoptosis index was measured by immunofluorescence analysis (Fig. 6c) in OE33 xenograft tissues by an antibody that only recognized cleaved caspase 3. Lapatinib plus foretinib combination therapy enhanced the apoptosis index by 3.38 fold compared to that of the control group (p = 0.048), by 4.96 fold compared to that of the lapatinib group (p = 0.025) and by 3.89 fold compared to that of the foretinib group (p = 0.032). Similarly, immunofluorescence analysis (Fig. 6d) of OE33 tumor xenografts with an antibody that recognizes carcinoma cells expressing the proliferative marker Ki-67, showed significantly lower number of carcinoma cells expressing Ki-67 per 100 total number of cells (proliferative index) in the lapatinib plus foretinib combination therapy group compared to that of the control, lapatinib or foretinib treatment groups. In the lapatinib plus foretinib treated group, the proliferative index (PI) was decreased by 55% (p = 0.0013), compared to the control group. Lapatinib or foretinib treatment alone didn’t show any reduction of the proliferative index or enhancement of the apoptosis index compared to those of the control. These results indicated that lapatinib plus foretinib combination therapy had stronger *in vivo* antiproliferative and apoptotic effects compared to lapatinib or foretinib single agent therapy.

### Effect of lapatinib and foretinib treatment on the survival of mice harboring esophageal adenocarcinoma

**Figure 7:**
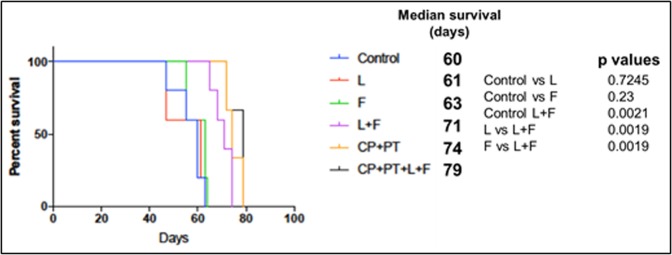
Improvement of animal survival by of lapatinib (L) and foretinib (F). OE33 cells (10 × 10^6^) were injected intraperitoneally in NOD/SCID mice and treatment started after 2 weeks with lapatinib and foretinib for 2 weeks. The curve represents the animal survival time from the beginning of therapy. Statistical group differences in survival time were calculated using logrank testing (GraphPad Prism 7.0).

We then evaluated the effect of lapatinib and foretinib on the survival of mice harboring OE33 peritoneal disseminated xenograft tumors as described by us earlier^22^. Kaplan-Meier curves of the different treatment groups and the comparison are shown in Fig. 7. The median survival of nonobese diabetic/severe combined immunodeficient (NOD/SCID) mice was 60 days in the control group. Lapatinib plus foretinib combination therapy prolonged the median animal survival from 60 days to 71 days (p = 0.0021). Lapatinib (61 days, p = 0.7245) or foretinib (63 days, p = 0.23) treatment alone didn’t show any significant survival advantage over control. The lapatinib plus foretinib combination therapy showed significant survival advantage not only over control, but also over lapatinib or foretinib treatment alone (p = 0.0019). Furthermore combining standard combination chemotherapy CP + PT with lapatinib (L) plus foretinib (F) showed an even more enhanced median animal survival of 79 days, (p = 0.0134 compared to control).

## Discussion

Targeted therapies with tyrosine kinase inhibitors are an emerging class of anticancer therapies that have shown promising clinical activity. Compared with other types of cancer, targeted therapy of EAC is still lagging behind^30^. EGFR, HER2 and MET are amplified and overexpressed in a subset of EAC patients, and they can be targeted in patients with EAC^14,31,32^. They are oncogenic drivers that signal for proliferation and survival. At present, the use of a monoclonal antibody against HER2, Trastuzumab/Herceptin, has been restricted to metastatic HER2-postive EAC^14^. Lapatinib, a dual EGFR and HER2 receptor tyrosine kinase inhibitor, has also been investigated in metastatic esophago-gastric adenocarcinoma but without much success^14,33^. Using only *in-vitro* studies, it has been suggested that MET activation may confer lapatinib resistance in EAC^31^. In this study, we verified this resistance *in-vitro* and, with lapatinib and foretinib treatment, we identified MET activation as a possible resistance mechanism to lapatinib in HER2 and MET co-activated EAC cells in *in-vivo* murine subcutaneous xenograft and peritoneal metastatic survival models of human EAC. In addition, we have shown induction of MET phosphorylation by stepwise inhibition of multiple ErbB family members. We found that the phosphorylation of MET protein was upregulated after stepwise exposure to increasing concentrations of lapatinib in OE19 cells indicating that the MET signaling pathway might be involved in the resistance of EAC to lapatinib.

EGFR and HER2 are tyrosine kinase receptors, and act as receptors for epidermal growth factor (EGF), a potent mitogen^34^. EGFR/HER2 showed frequent expression in EAC and influenced patient survival^35^. Despite a strong clinical response to the HER2 inhibitor in some HER2-positive esophagogastric cancer patients, some HER2-positive patients did not show significant clinical benefits^36^, and alternative growth signaling pathways were suggested in HER2-inhibitor resistance mechanisms^14^. It has been reported that *In-vitro* targeting of HER2 in EAC cell lines with lapatinib resulted in inhibition of the phosphorylated HER2 receptor and downstream signaling with the development of lapatinib resistance due to MET amplification^31^. Similarly in our study, signaling through alternative growth pathways like MET were noted to produce HER2-targeted lapatinib resistance. Though these *in-vitro* studies shed some insight into the mechanism of lapatinib resistance, they may not represent the situation *in-vivo*. Also, *in-vitro* experiments are not enough for pre-clinical development of any novel combination therapies. For translation of pre-clinical experiments to clinical trials *in-vivo* experiments are indispensable.

We show that lapatinib is a potent inhibitor of HER2 phosphorylation and foretinib is a potent inhibitor of MET phosphorylation in EAC cells. We found that cell lines with HER2 activation had the best response to lapatinib, whereas cell lines with MET activation showed the best response to foretinib. Contrary to that, OE19 EAC cells without MET phosphorylation but with MET expression were sensitive to foretinib. Foretinib is a multi-kinase inhibitor targeting multiple receptor tyrosine kinases (RTKs) implicated in cancer cell proliferation, and its mechanism of action is incompletely understood^37^. Its effect on OE19 cells may be due to hitherto unidentified mechanisms. HER2 phosphorylation in HER2 driven OE19 EAC cells rendered OE19 cells more sensitive to single-agent lapatinib induced cell growth inhibition and apoptosis than that of foretinib. Similarly, MET phosphorylation in MET driven ESO51 EAC cells rendered ESO51 cells more sensitive to single-agent foretinib induced cell growth inhibition and apoptosis than that of lapatinib. In addition, lack of enhanced growth inhibition and apoptosis was observed when lapatinib was combined with foretinib in OE19 and ESO51 EAC cells. HER2 and MET co-activation rendered OE33 EAC cells less sensitive to single-agent lapatinib or foretinib induced cell growth inhibition and apoptosis, but enhanced growth inhibition with apoptosis was noted when combining lapatinib with foretinib. Interestingly, we found that foretinib consistently increased both pHER2 and HER2 expressions in OE33 cells, but not in OE19 cells. This effect could be due to preferential killing of OE33 cells with MET activation by foretinib with a resultant increase in the OE33 cell population having HER2 activation/overexpression. Thus this study also supports our hypothesis that in EAC cells where MET and HER2 are co-expressed, these two receptors can work together to prevent cell apoptosis and enhance cell growth.

Molecular mechanisms of lapatinib resistance have been studied extensively in many cancers including esophageal adenocarcinoma^31,38,39^. But in clinical trials lapatinib failed to show survival benefit in HER-2 positive esophageal cancer. Therefore, to predict which patients will more likely to get a benefit from combination therapy is of great interest. This study has the advantage of *in-vivo* targeting of HER2 and MET pathways by using FDA approved, clinically utilized small molecule inhibitors. Our subcutaneous xenograft studies evaluated comparative antitumor effects of lapatinib and foretinib monotherapy as well as combination therapy in HER2 and MET co-activated OE33 EAC tumor xenografts. This study clearly demonstrated the advantage of *in-vivo* targeting of HER2 and MET pathways by lapatinib and foretinib combination over single agent therapy. Combining lapatinib with foretinib significantly decreased net tumor volume, relative tumor volume (RTV) and average tumor weight without effecting mice average weight over single agent therapy. This enhanced antitumor effect could indicate important clinical implications. The combination of anti-MET therapy with anti-HER2 therapy could have a direct clinical benefit in a subset of EAC patients. Further investigation of the mechanisms of the antitumor activity of lapatinib plus foretinib combination therapy, compared to lapatinib or foretinib monotherapy, by immunohistochemical analyses of tumor tissues revealed a significantly reduced Ki67-proliferative index, and also a significantly enhanced cleaved caspase 3-apoptosis index. This effect of enhanced apoptosis with combination therapy was further confirmed by western bot analysis. Similar to *in-vitro* effects, we found that the *in-vivo* use of lapatinib or foretinib caused a decrease in phosphorylation of HER2 and MET. Thus, these signaling pathway changes are likely present not only *in-vitro*, but also *in-vivo*. These markers might therefore represent valid markers of *in-vivo* activity and warrant clinical validation.

In addition, we assessed a possible survival benefit of lapatinib or foretinib alone and as a combination therapy. Different subcutaneous as well as orthotopic mouse xenograft models have been described for *in-vivo* assessment of anticancer drugs in EAC^40–42^. However, these subcutaneous implantation models do not represent a patient environment and have been shown to rarely metastasize. A better model would be an orthotopic EAC model. Unfortunately, it is very difficult to establish a mouse orthotopic EAC model, and its reproducibility remains challenging. Furthermore, an invasive procedure is required that induces inflammation and therefore may inadvertently influence subsequent therapeutic interventions. Because of this, we used a simple, less invasive, and more patient-like EAC survival model as published before^22^. Our results showed that combination of lapatinib with foretinib significantly increased mouse survival over lapatinib or foretinib alone. This survival benefit was further enhanced when lapatinib plus foretinib was combined with the standard chemotherapeutic agents carboplatin plus paclitaxel. Our data demonstrates that the combination of lapatinib plus foretinib did inhibit tumor progression in an OE33 experimental EAC model with co-activation of HER2 and MET. Therefore, combing lapatinib with foretinib appears to represent a new targeted therapy regimen in HER2 and MET co-activated EAC.

In conclusion, our results showed that lapatinib plus foretinib combination therapy had increased antitumor activity. The increased antitumor activity resulted in prolonged animal survival in OE33 experimental EAC. This enhanced antitumor activity supports clinical evaluation of this targeted combination therapy as a personalized medicine approach in EAC.

### Ethics statement

All mouse experiments used in this study were carried out in accordance with the standards and guidelines of the Institutional Animal Care and Use Committee (IACUC) at the University of Notre Dame, and confirmed to NIH guidelines. All animal research used in this study were approved by the University of Notre Dame IACUC under protocol 15-08-2631. At the end of the experiments, mice were euthanized by CO2 using the Euthanex Euthanasia chamber according to University of Notre Dame IACUC-approved procedures.

## Supplementary information

Supplementary Information
