# Supplementary material for: Combination effect of lapatinib with foretinib in HER2 and MET co-activated experimental esophageal adenocarcinoma
Source: Sci Rep. 2019 Nov 26;9:17608. doi: 10.1038/s41598-019-54129-7 (PMC6879590; doi:10.1038/s41598-019-54129-7)

Title

Combination effect of lapatinib with foretinib in HER2 and MET co-activated experimental esophageal adenocarcinoma

Authors

Md Sazzad Hassan[1, 4, #], Fiona Williams[2], Niranjan Awasthi[1, 4], Margaret A. Schwarz[4, 5], Roderich E. Schwarz[1, 4], Jun Li[7] and Urs von Holzen[1,3,4,6]

Supplemental Information

Figure S1

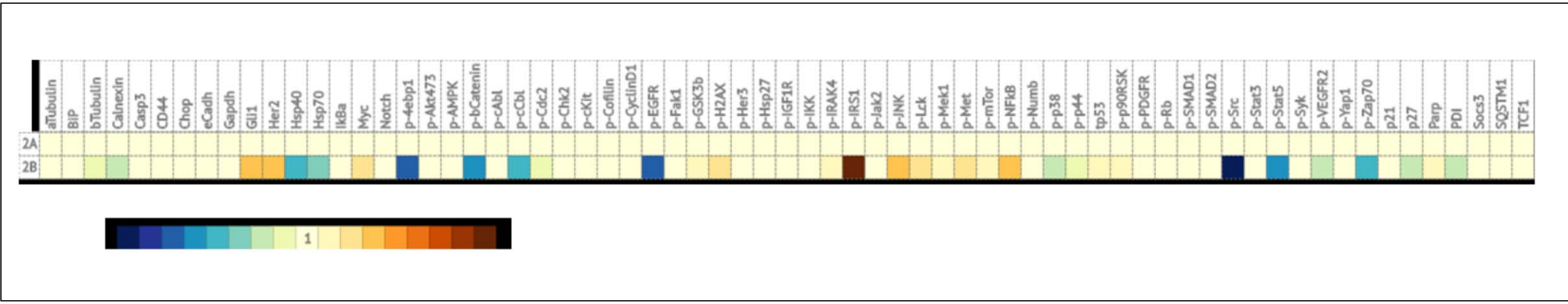

Figure S1: Immuno-paired antibody detection of altered protein expression and signaling pathways in LPR-OE19 relative to OE19. Analysis was performed using IPAD technology by ActivSignal, Inc. The heatmap represents changes of protein expression in LPR-OE19 (2B) over OE19 (2A). Two biological replicates were analyzed per condition.

Original images

Figure 1

pMET

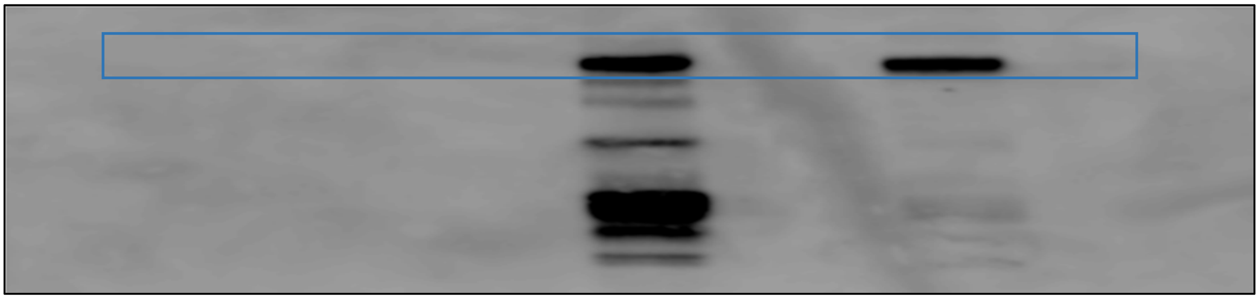

MET

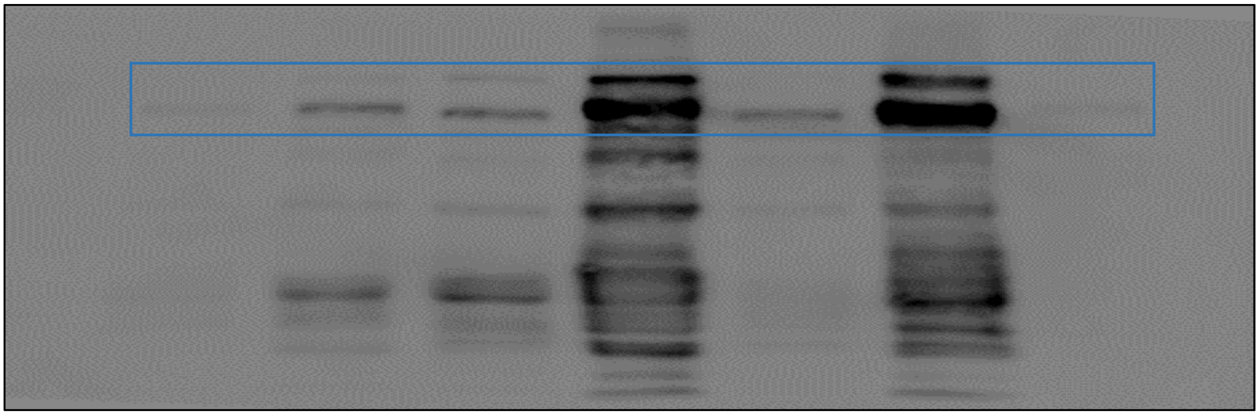

pHER2

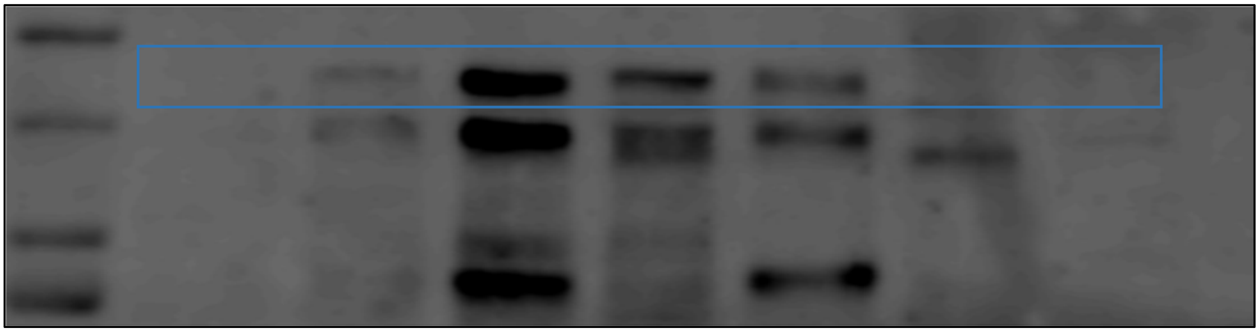

HER2

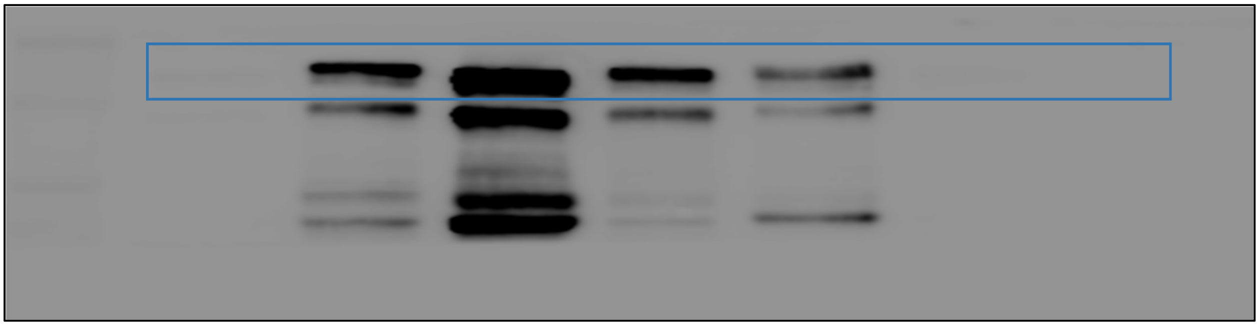

β-actin

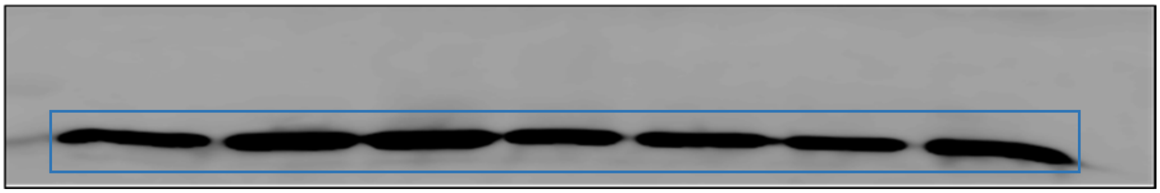

Figure 3b

pMET (multiple exposures)

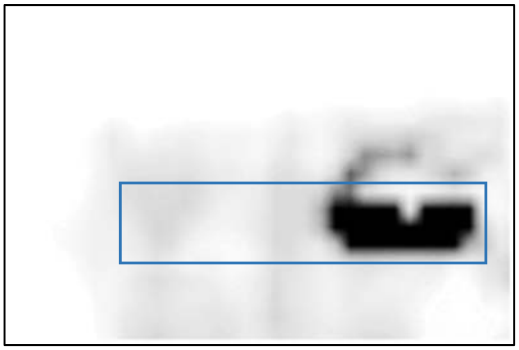

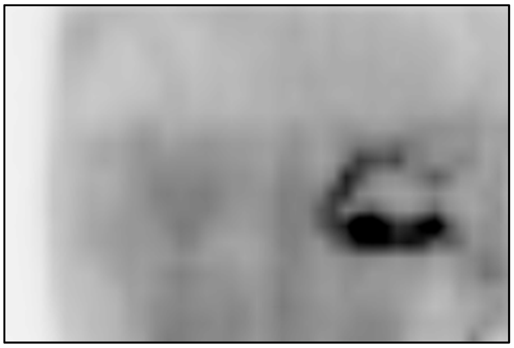

MET (multiple exposures)

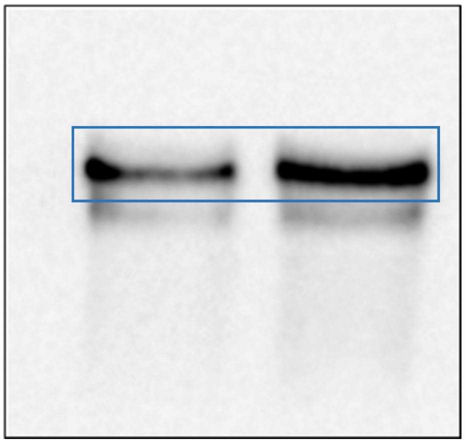

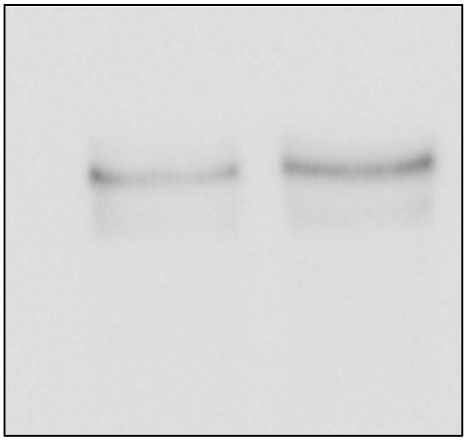

pHER2

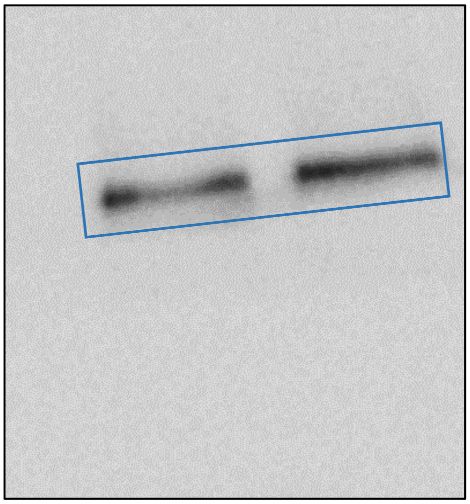

HER2 (multiple exposures)

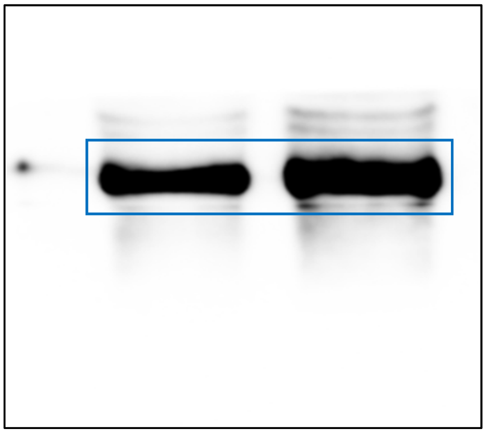

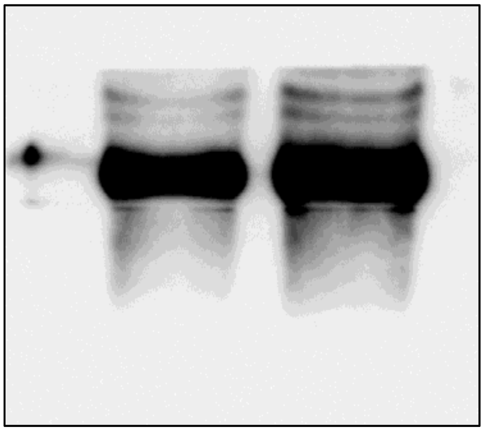

β-actin

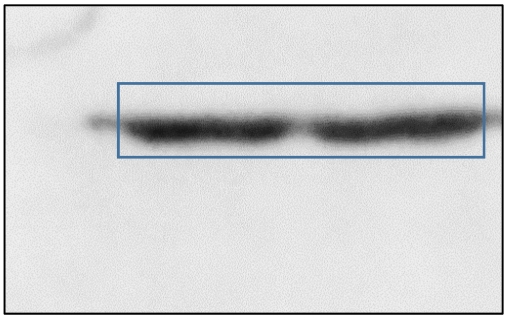

Figure 4a

pMET

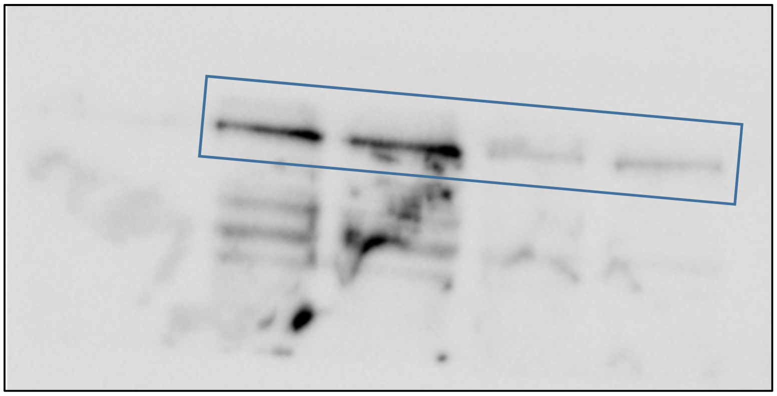

MET (multiple exposures)

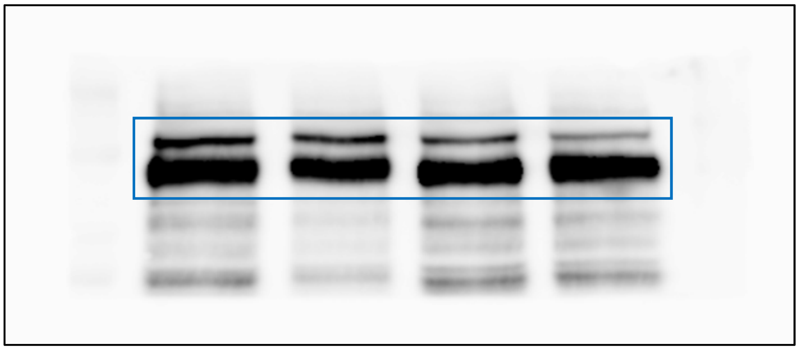

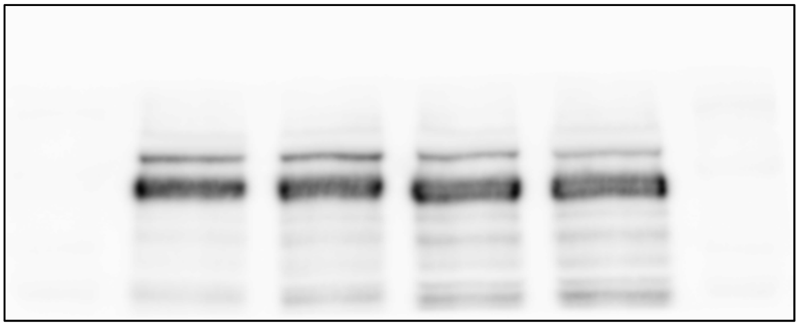

pHER2

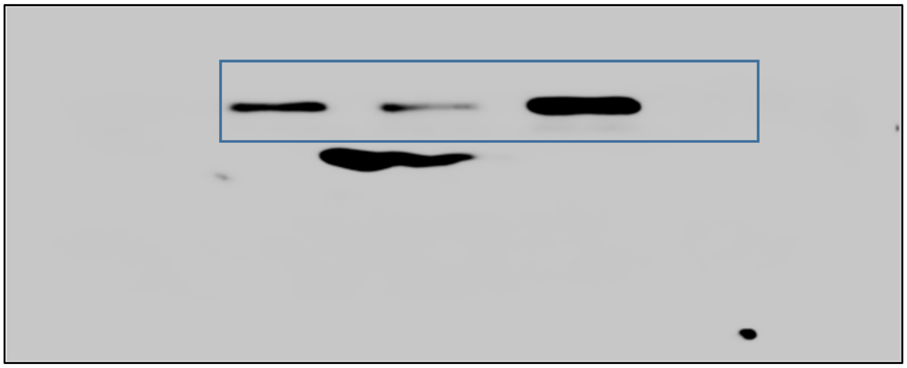

HER2

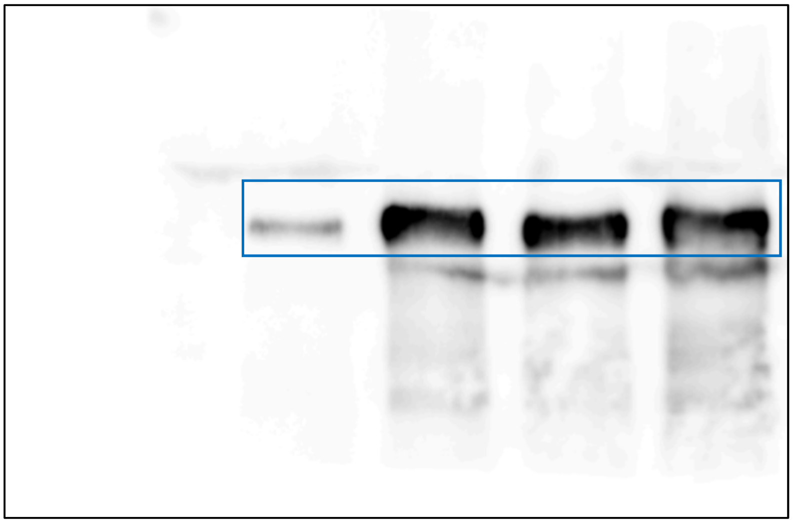

Cleaved PARP

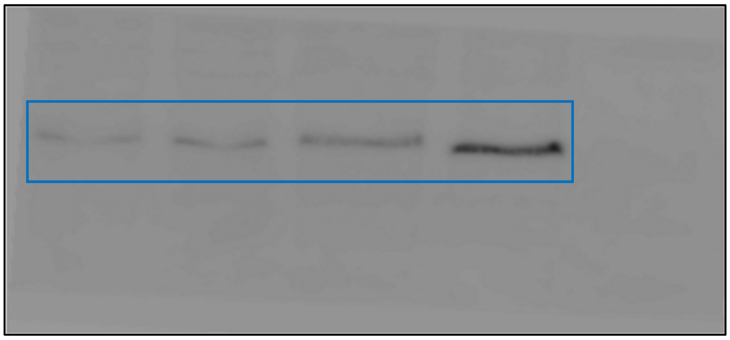

Cleaved caspase 3

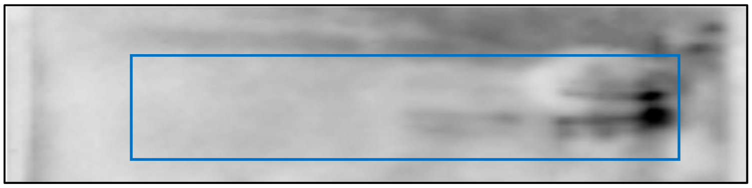

β-actin

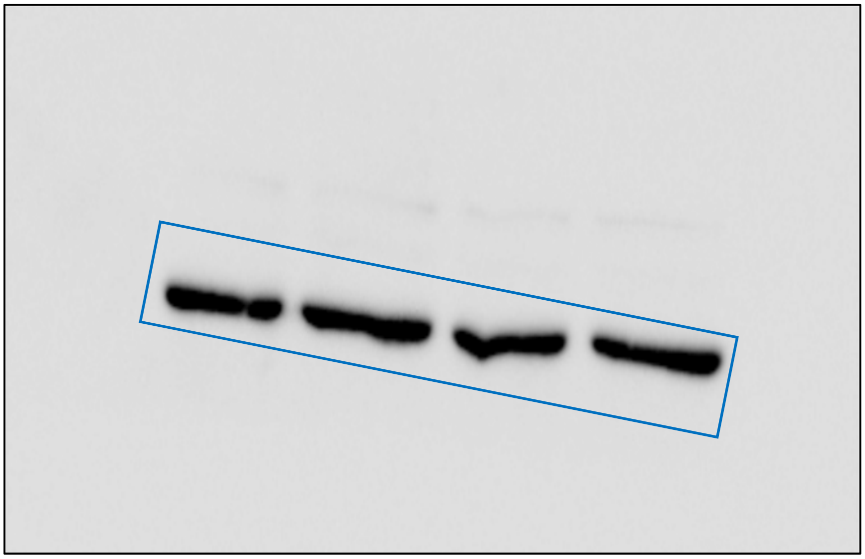

Figure 4b

pMET

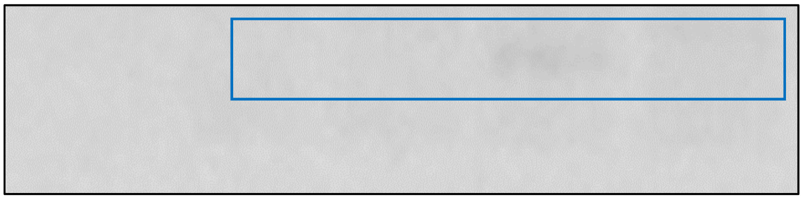

MET

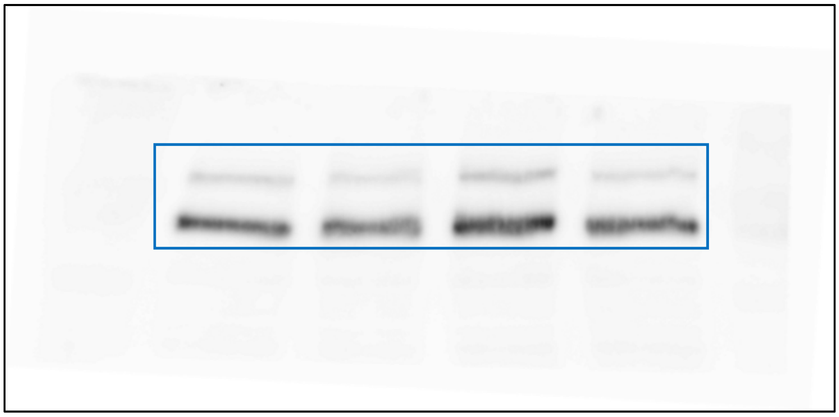

pHER2

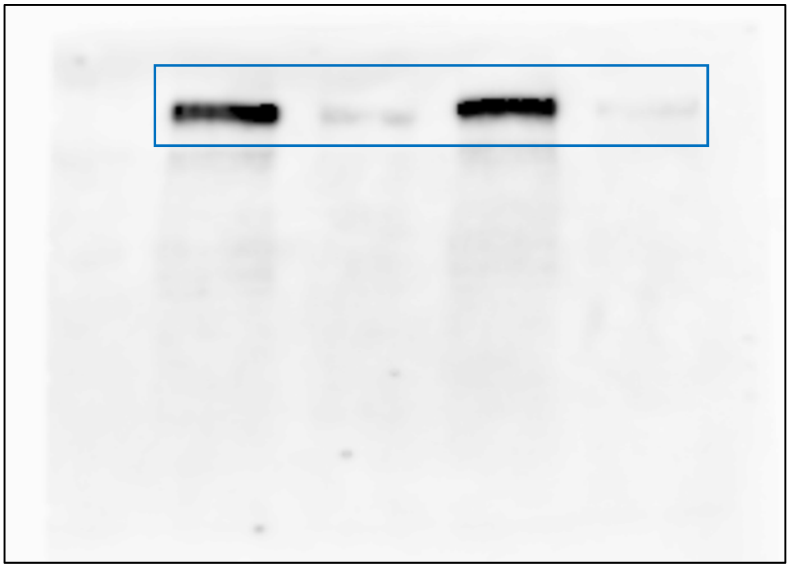

HER2 (multiple exposures)

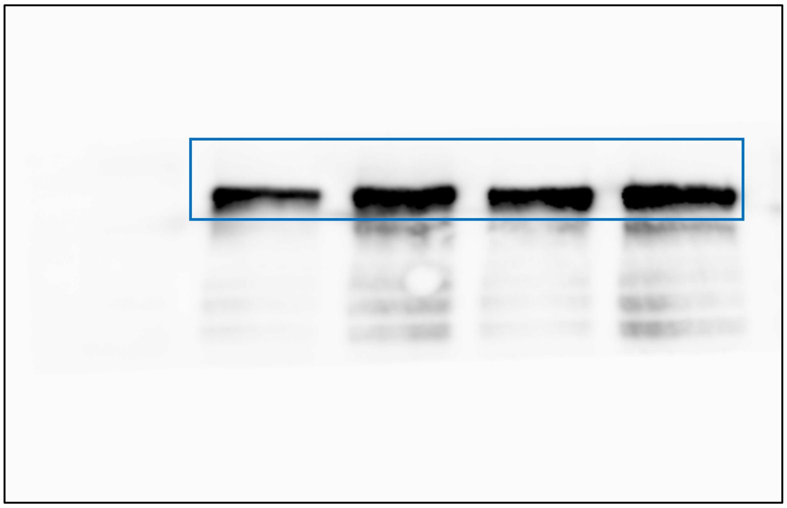

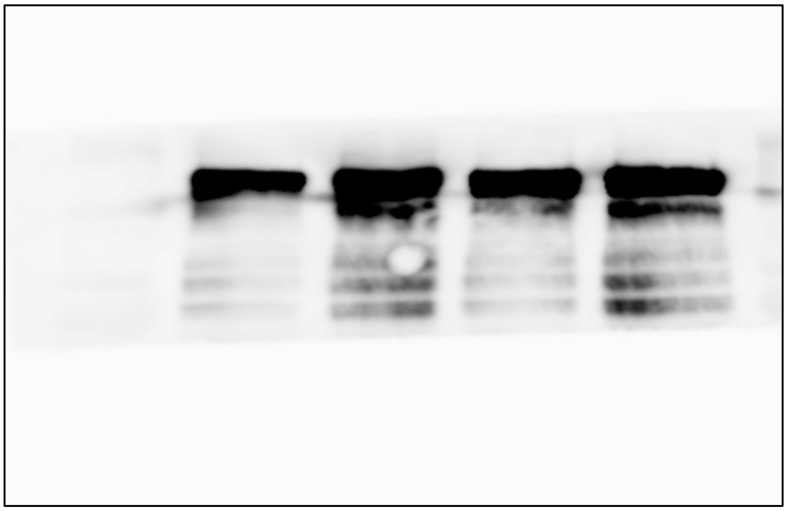

Cleaved PARP (multiple exposures)

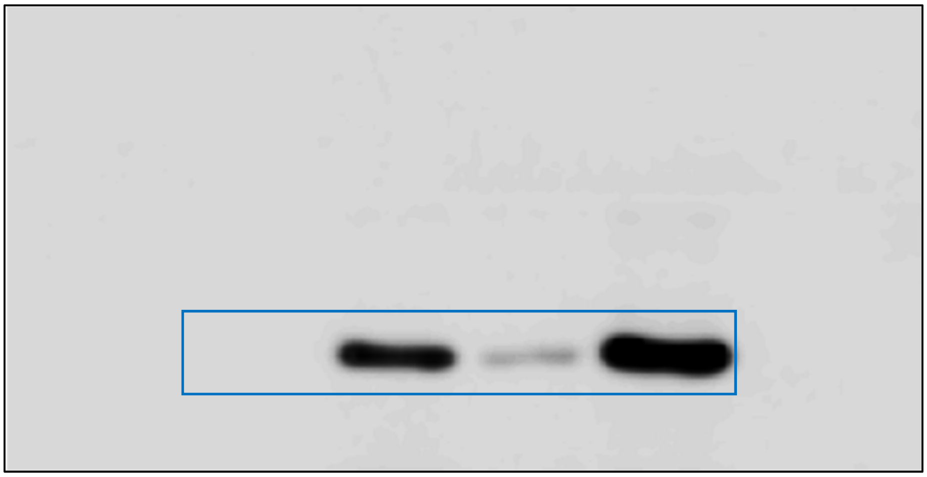

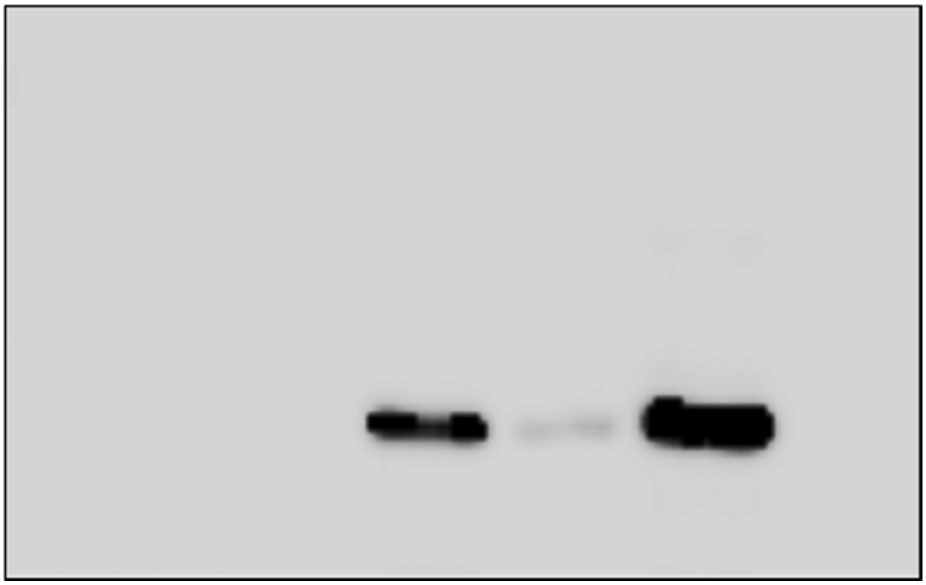

Cleaved caspase 3

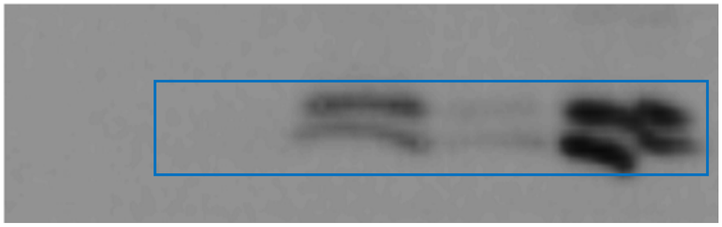

β-actin (multiple exposures)

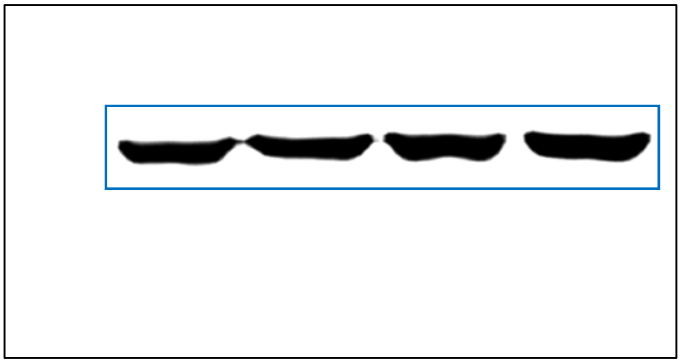

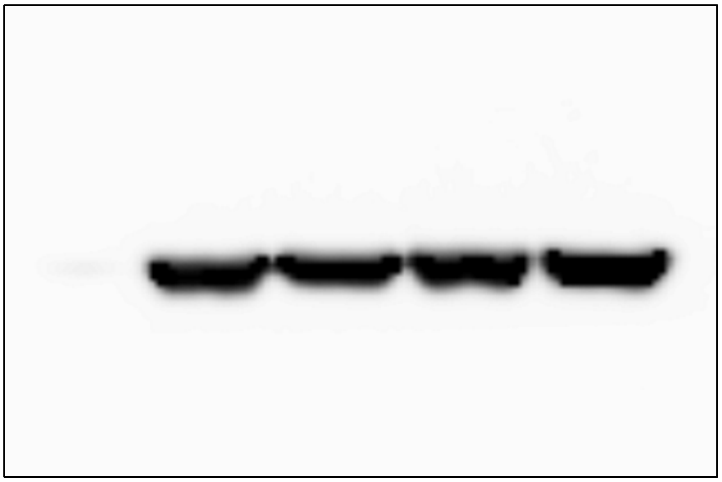

<u>**Figure 4c**</u>

PMET (multiple exposures)

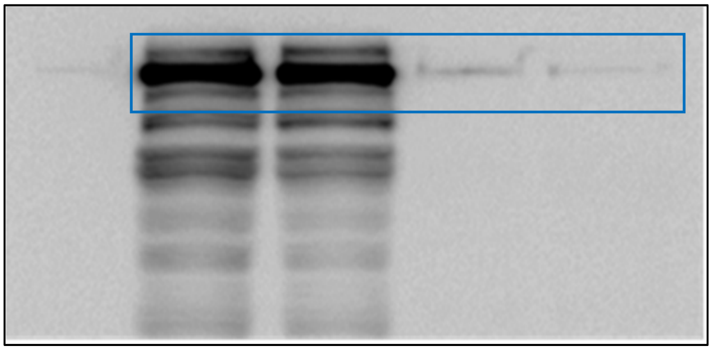

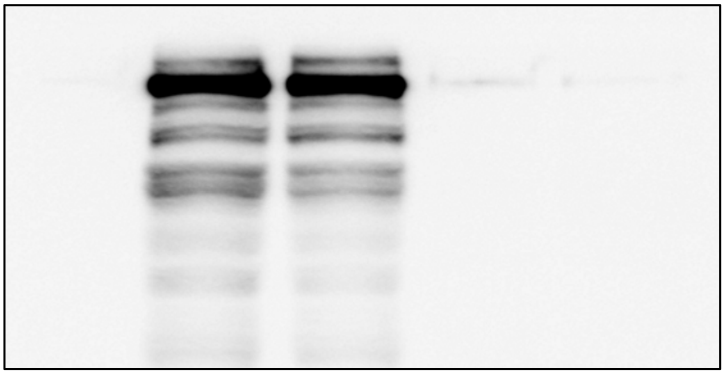

MET

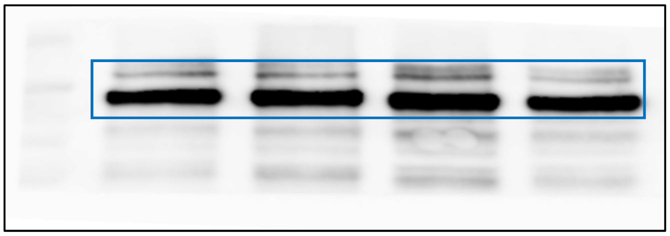

pHER2

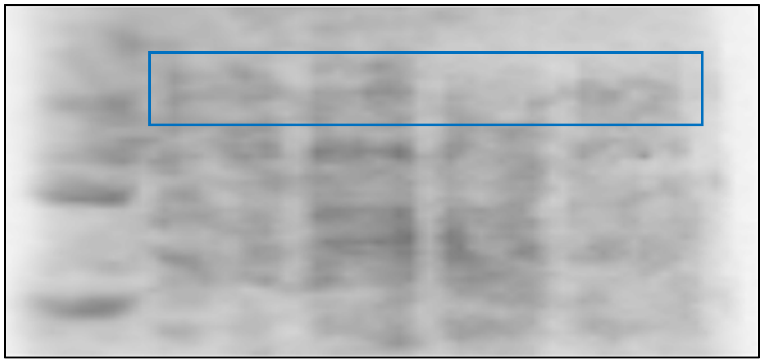

HER2

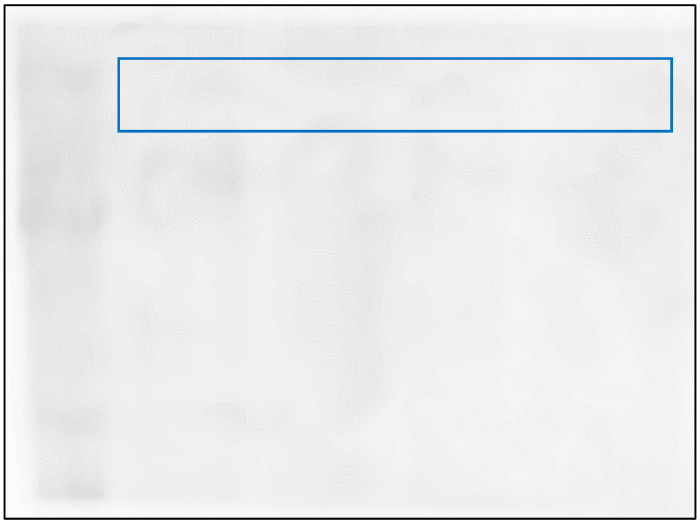

Cleaved PARP

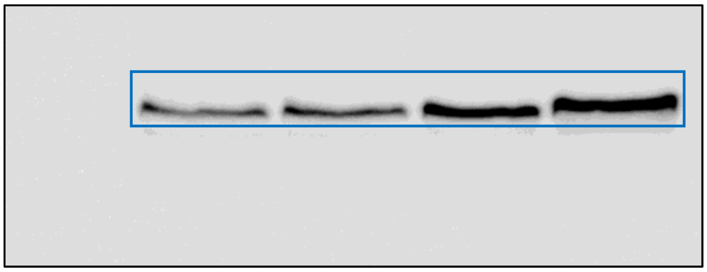

Cleaved caspase 3

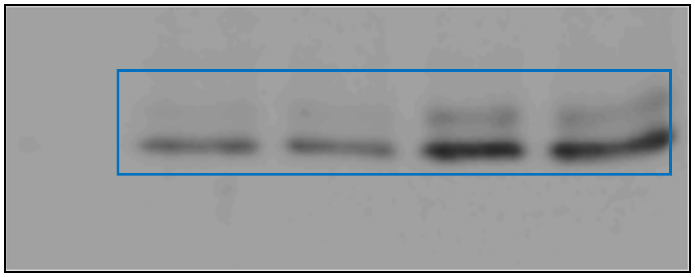

β-actin (multiple exposures)

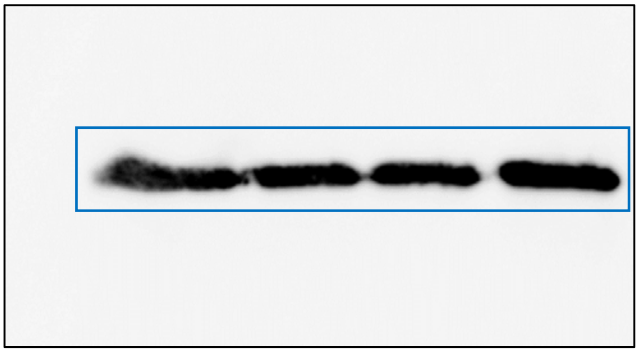

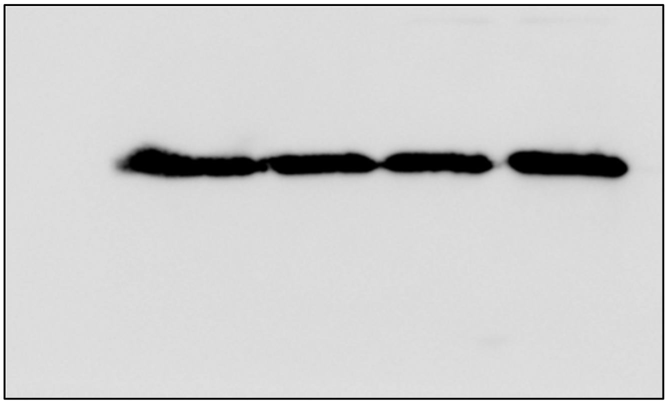

Figure 6a

pMET

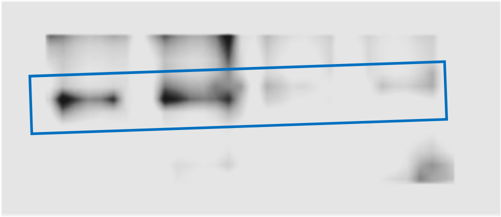

MET

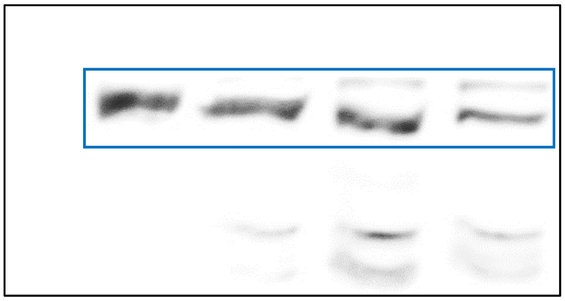

pHER2

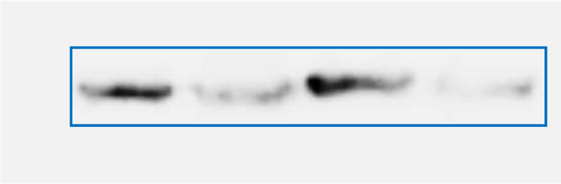

HER2

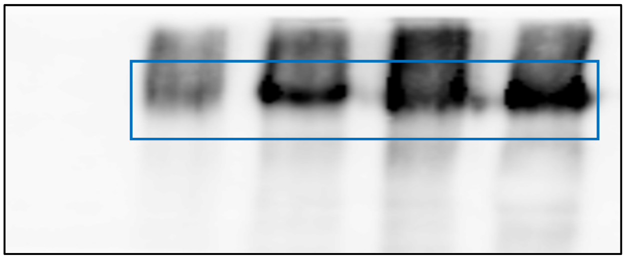

C-PARP

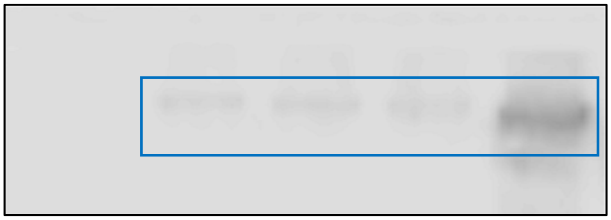

C-caspase 3

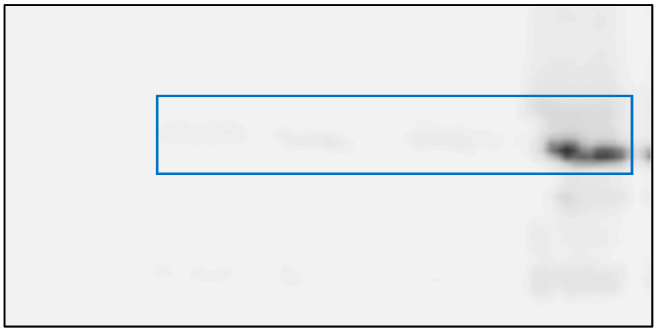

β-actin

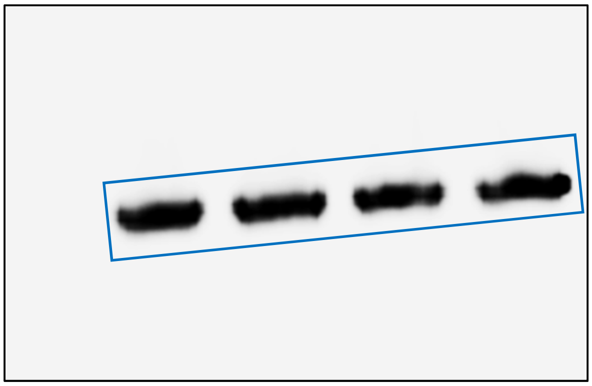

Supplement: Supplementary file 1 — Supplementary Information [file 41598_2019_54129_MOESM1_ESM.pdf]
